# Early assessment of clinical complexity and home care in patients affected by trisomy 13 and 18

**DOI:** 10.1007/s00431-025-06020-z

**Published:** 2025-02-12

**Authors:** Anna Zanin, Matteo Patti, Isabella Rosato, Antuan Divisic, Francesca Rusalen, Irene Maghini, Caterina Agosto, Franca Benini

**Affiliations:** 1 Palliative Care and Pain Service, Department of Women’s and Children’s Health, University of Padua, Via Giustiniani 3, 35128 Padua, Italy; 2 Department of Women’s and Children’s Health, University of Padua, Padua, Italy; 3 Unit of Biostatistics, Epidemiology and Public Health, Department of Cardio-Thoraco-Vascular Sciences and Public Health, University of Padua, Padua, Italy

**Keywords:** Neonatology, Trisomy 13, Trisomy 18, Home care, Pediatric palliative care

## Abstract

**Purpose:**

Trisomy 13 and 18 consist of a recurrent pattern of multiple congenital anomalies. The aim of this study was to analyze the clinical characteristics and disease trajectory of a cohort of children with trisomy 13 and 18 followed up by an Italian pediatric palliative care service.

**Methods:**

A single-center retrospective observational study was conducted examining the medical records of patients with trisomy 13 and 18 seen in the Pediatric Palliatives Care (PPC) center of the University Hospital of Padua from 2007 to 2022.

**Results:**

Seventeen patients were included in the analysis. All were born alive; four children are still alive and only three (23%) died at home. All presented high care complexity, as estimated by ACCAPED index (median 86, range 38–129). The median time to receive care from PPC was 3 months (0–108). All patients’ parents shared an advance care plan with the PPC team: 13/17 patients (76%) accepted a do not resuscitate (DNR) order. Approximately 12% of patients received at least one surgery. The trend of survival compared with other cohorts reported in the literature does not appear to differ significantly after the initial stages.

**Conclusions:**

The possible recognition of an early evolution toward medical complexity and the availability of home care resources and programs are crucial factors in the management of these children. These indices could become a driving factor in the definition of new outcomes that are more patient-oriented, in addition to mortality.

**Table:** 

**What is known:** *• Trisomy 13 and 18 are serious genetic conditions with high mortality rates. In the last years medical interventions including surgery are being offered more frequently, though the appropriateness of these interventions is still debated.* **What is new:** *• The study emphasizes the crucial role of early referral to specialized pediatric palliative care teams and the coordination they provide enabling families to care for their children at home, even with complex medical needs.*

## Introduction

Trisomy 18 (T18) and trisomy 13 (T13) result from an extra 18 and 13 chromosome. They are the second and third most common autosomal trisomies in live births, after trisomy 21 [1, 2]. Both syndromes consist of a recurrent pattern of multiple congenital anomalies, in particular congenital heart disease (CHD), which is present in approximately 38% and 45% of patients with T13 and T18 [3]. Common causes of death are central apnea or end-organ dysfunction, such as pulmonary hypertension or heart failure (HF). One-year mortality rate for this diagnosis is nearly 90% [4, 5].

In the past, T13 and T18 were considered fatal anomalies, and palliative care was offered as the only possibility for these children and their families [6, 7]. Recently, Kosho et al. showed that medical intervention, including ventilation, surgeries, and intensive care treatments, improved the 1-week and 1-year survival rates compared with the existing population studies [8]. Many other studies suggest an increase in the frequency of surgeries in these patients, indicating a shift in the treatment strategy [4, 9], although the appropriateness of surgical treatment options for children with T13 and T18 continues to be debated [10]. In these patients, there is broad variability of cardiac lesions and non-cardiac comorbidities, which means a set rule for cardiac surgical candidacy cannot be made [3]. The care for these children and their families is complex [3]. Pediatric palliative care (PPC) teams offer longitudinal presence across care settings to families regardless of whether or not they choose to pursue medical interventions for their child, assisting the family in understanding the child’s comorbidities and making medical decisions, since the evolution can be unpredictable [11, 12]. PPC provides symptom management for the patient and psychological support to families who choose comfort care for their child, both during the child’s life and after death [13, 14]. The objective of this study is to increase the knowledge regarding the experiences of children with T13 and T18 with palliative care services, using data from an Italian cohort.

## Material and methods

A single-center observational retrospective study was conducted reviewing all medical charts of patients with T13 and T18 referred to our PPC center of the Tertiary Care University Hospital of Padua. The study included inpatient and outpatient data of patients born between January 2007 and December 2022. Patients included in the sample were assessed using ICD-9 and ICD-10 coding, as defined in the literature. The study was conducted in accordance with the Declaration of Helsinki, and the protocol was approved by the Institutional Review Board (number n 314n/AO/22).

For each patient, we analyzed socio-demographic and clinical characteristics (gender, genetic diagnosis, alive at birth, gestational age (GA), birth weight (BW), months followed by the PPC, age at time of study) and end-of-life care for patients who died (cause of death, place of death, age of death). To describe the degree of complexity of care, we listed some specific clinical aspects, including number of medical devices, number of daily drugs administration/24 h, polypharmacy defined as the concurrent use of five or more medication items by one individual [15, 16], number of hospitalization in the last year, number of surgical procedures performed, and do not resuscitate order (DNR). We also used two scores: the Assessment of Complex Clinical Assistance Needs in Paediatrics (ACCAPED) [17] and The Family Reported Outcome Measure (FROM-16) [18]. The first is a tool which contains information on a range of clinical issues (breathing, nutrition, epilepsy and state of consciousness, skin and tissue integrity, mobility, ability to communicate, sleeping characteristics, fecal continence, medications, pain, and unexpected or unpredictable events that could lead to death) to detect the complexity of clinical needs and the allocation to the appropriate PPC care level [17]. The FROM-16 is a questionnaire that measures the impact on the quality of life (QoL) of an adult family member [18] which is normally the principal caregiver of the child; this was recorded by medical charts and reported for children still alive. The questions in FROM-16 are divided into two domains: emotional (6 questions, maximum score of 12) and personal and social life (10 questions, maximum score of 20).

The characteristics of this cohort were presented using median and range for continuous variables and absolute and relative frequencies for categorical variables. Comparisons between different groups were analyzed using Fisher’s exact test for categorical variables and the Mann–Whitney test for continuous variables. A Kaplan–Meier survival curve was created for patients with T18 included in the study. Individual patient data from published Kaplan–Meier survival curves were reconstructed using R package IPDfromKM [19]. We considered the survival curves created for three cohorts of patients with T18: the first were an English [20] and a Portuguese [6] cohort; the third one was a large Canadian cohort in which individuals with T18 were identified using linked health administrative databases [10]. The Italian cohort survival curve for patients with T18 was compared with the reconstructed survival curves using Gehan-Breslow’s test. Analyses were performed using the R Statistical Software (R Core Team (2022) Vienna, Austria.). A *p* value < 0.05 was considered statistically significant.

## Results

**Fig. 1:**
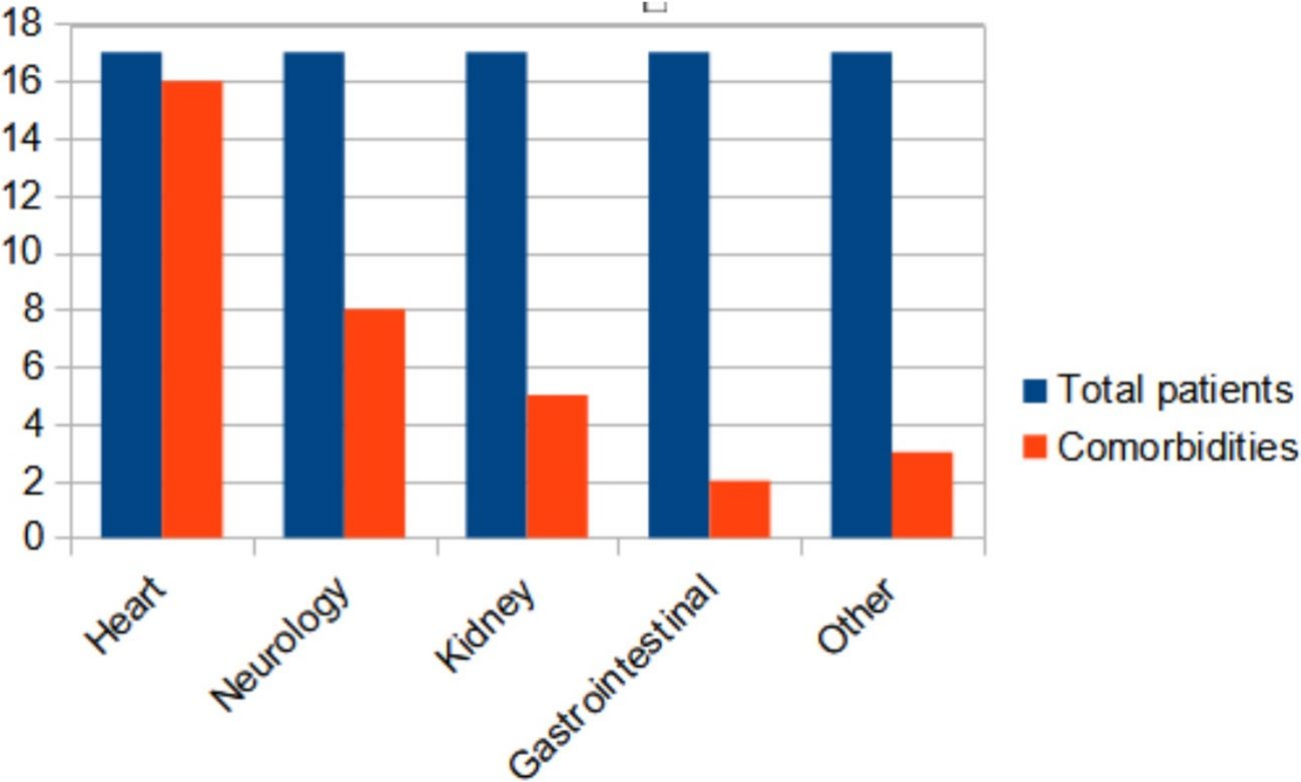
Congenital conditions at birth, grouped accordingly into different systems (blue = total patients, red = comorbidities)

Over the study period from January 2007 to December 2022, our PPC team followed 17 children in this cohort, 3 of whom had T13 and 14 with T18 (Table 1). All of them were alive at birth, median GA was 37.1 weeks (range 32.8 weeks to 41.7 weeks, mean value 36.2 ± 1,6). Median BW was 1775 g (mean 1867 g ± 469 DS), and median time of follow up done by the PPC was 3 months (0–108, mean value was 15 months ± 28 SD). Only five patients had a prenatal diagnosis; six patients received neonatal resuscitation and among this group, only one had a prenatal diagnosis. Specific heart defects and other comorbidities are summarized in Fig. 1 and Table 2. Among non-cardiac structural defects, agenesis of corpus callosum was the most common (18.75%) (Table 2). Data on mosaicism were not available. In one case (5.9%), the trisomy T18 was also associated with Krabbe disease in consanguineous parents.

**Table 1:** Descriptive analysis of the Italian cohort’s characteristics stratified according to diagnosis

			Trisomy 13 *N* = 3	Trisomy 18 *N* = 14	Full cohort *N* = 17
Gender (%)	F M		1 (33.3%) 2 (66.7%)	9 (64.3%) 5 (35.7%)	10 (58.8%) 7 (41.2%)
Mean (SD) gestational age (range, week), *n* = 15			35.6 (± 3.3)	35.4 (± 1.5)	36.2 (± 1.64)
Median gestational age (range, week), *n* = 15			35 (33–38)	37 (32–41)	37 (32–41)
Mean (SD) birth weight (range, g), *n* = 13			2140(± 96)	1845(± 483)	1867(± 469)
Median birth weight (range, g), *n* = 13			2140 (2140–2000)	1845 (1045–2770)	1775 (1045–2770)
Median percentile birth weight, *n* = 13			15th	< 3rd (< 3rd–75th)	< 3rd (< 3rd–75th)
Median time (months) followed by palliative care (range), *n* = 17			2.0 (0.1–12)	5.5 (0–108)	3.0 (0–108)
Alive at time of study (%)			0 (0.0%)	4 (28.6%)	4 (24%)
Median age at time of study (range), *n* = 4			-	6 (1–9)	6 (1–9)

**Table 2:** Non-cardiac congenital defects or additional conditions at birth (individual data for each patient) (*ACC* agenesis of corpus callosum, *CPCs* choroid plexus cysts, *CVH* cerebellar vermis hypoplasia)

Patient	Neurological	Gastrointestinal	Renal	Other
1	ACC	-	-	Sdr Krabbe
4	CPCs	-	-	
5	-	-	Horseshoe kidney	
6	Hydrocephalus	Hepatomegaly	Duplex kidney	Pulmonary hemorrhage
7	-	-	Renal agenesis	
8	ACC	-	-	
9	ACC	-	-	Ocular defects
10	CVH	-	-	
13	CPCs	Volvulus	Horseshoe kidney	
16	CVH	-	Megaureter	
TOT (%)	8/17 (47.05%)	2/17 (11.76%)	5/17 (29.41%)	3/17 (17.64%)

Table 3 shows the cause of death in both groups of patients and the location of death. Most patients (7/13, 54%) died in the delivery room (first 24 h of life) or in pediatric wards due to readmission after being discharged from the Neonatal Intensive Care Unit. The remaining patients died in the Pediatric Hospice (3/13, 23%) or at home (3/13, 23%). In all three latter cases, home-care support has been delivered by the PPC team to the family who asked to keep the baby at home at the end of life. Median age of death was 2 months (0–17), while the mean value was 2.9 months (± 5.03). The most frequent underlying factors associated with death were respiratory failure and HF.

**Table 3:** End-of-life characteristics: causes, locations, and ages of death

		Trisomy 13 *N* = 3	Trisomy 18 *N* = 10	Total *N* = 13
Cause of death (%), *n* = 13	Cardiac arrest Respiratory insufficiency Missing	1 (33.3%) 2 (66.7%) -	1 (11.1%) 8 (88.9%) 1	2 (17%) 10 (83%) 1
Place of death (%)	Domicile Hospice Hospital	1 (33.3%) 1 (33.3%) 1 (33.3%)	2 (20%) 2 (20%) 6 (60%)	3 (23%) 3 (23%) 7 (54%)
Median age (months) at death (range)		2 (0.1–13)	2.5 (0–17)	2 (0–17)
Mean age (months) at death (SD)		5 (± 7.7)	5.5 (± 5.8)	5.21 (± 5.81)

The complexity of care for all children in the study was assessed based on the number of devices and daily medications required (Table 4), as well as the ACCAPED calculation [15]. A score greater than 50 on the ACCAPED scale was used as an inclusion criterion for specialized PPC [15]. All these patients required tertiary-level PPC coordination, with a median ACCAPED score of 86 (range: 38–129). This complexity of care has an impact also on family organization, as seen in that for 76.5% of families, only the father was employed, and mothers most were considered as principal caregivers. The median number of medical devices was 3, which was higher in alive patients, compared to deceased patients (4.5, range 2–7); polypharmacy was detected in 76.5% with a statistically significant higher number of medications in the deceased cohort of patients (Table 4). Four of our patients were discharged home with O_2_, one with CPAP ventilation, one with high flow support, and two patients had tracheostomy with use of mechanical ventilation > 16 h per day. Half of alive patients were hospitalized more than once in the last year, and 69.2% of the patients alive at the time of the study reported more than one hospitalization in the last year of life. All patients shared an advance care plan with the PPC team: 13/17 patients (76%) agreed for a DNR (Table 5).

**Table 4:** Descriptive analysis of the Italian cohort’s complexity of care

		Italian cohort
Median number of medical devices (range), *n* = 16		3 (0–5)
Median number of drugs taken daily (range), *n* = 8		4 (1–10)
Polypharmacy (%), *n* = 8	Yes No	3 (38%) 5 (63%)
Median ACCAPED (range), *n* = 17		86 (38–129)
Median number of hospitalizations in the last year (range), *n* = 15		1 (0–12)
Median number of procedures performed (range), *n* = 17		0 (0–3)
DNR (%), *n* = 17	Yes No	13 (76%) 4 (23%)
Median FROM 16 Emotional (range), *n* = 3		1 (0–4)
Median FROM 16 Personal (range), *n* = 3		12 (11–12)

Polypharmacy described as ≥ 5 drugs/daily; medical device list includes aspirator, nasogastric tube, tracheostomy, enteral nutrition pump, pulse oxymeter

*ACCAPED* scheda di ACcertamento dei bisogni Clinico Assistenziali complessi in PEDiatria, *DNR* do not resuscitate order, *FROM-16* Family Reported Outcome Measure-16

**Table 5:** Complexity of care of alive and deceased patients

		Total *N* = 17	Alive *N* = 4	Deceased *N* = 13	*p* value*
Median number of medical devices (range), *n* = 16		3 (0–7)	4.5 (2–7)	3 (0–5)	0.248
Number of drugs taken daily (*n*, %), *n* = 17 0 1–3 4–6 7–10		9 (53%) 3 (18%) 3 (18%) 2 (12%)	1 (25%) 0 (0%) 1 (25%) 2 (50%)	8 (62%) 3 (23%) 2 (15%) 0 (0%)	0.039
Polypharmacy (n, %), *n* = 17	Yes No	3 (18%) 14 (82%)	2 (50%) 2 (50%)	1 (8%) 12 (92%)	0.052
Median ACCAPED (range), *n* = 17		86 (38–129)	86 (38–129)	87 (38–104)	0.955
Patients who were hospitalized 1 + times in the last year (*n*, %), *n* = 15	Yes No	10 (66.7%) 5 (33.3%)	2 (50%) 2 (50%)	8 (73%) 3 (27%)	0.409
DNR (%), *n* = 17	Yes No	13 (76%) 4 (23%)	2 (12.5%) 2 (12.5%)	11 (85%) 2 (15%)	0.22

Polypharmacy described as ≥ 5 drugs/daily, medical devices

*ACCAPED* scheda di ACcertamento dei bisogni Clinico Assistenziali complessi in PEDiatria, *DNR* do not resuscitate order, *FROM-16* Family Reported Outcome Measure-16

^a^Mann-Whitney test or Fisher’s exact test according to variables investigated

Four patients with trisomy 18 are still alive with a median age at time of study of 6 years. For three of them, we were able to report the score obtained on the FROM-16 [16]. Median score for FROM-emotional score was 1 out of 12 (range 0–4 out of 12) and FROM-personal score was 12 out of 20 (range 11–12 out of 20), with a total of 13/34 (12–15). The maximum score is 32, with a higher score indicating a greater impact on the family member’s QoL.

**Fig. 2:**
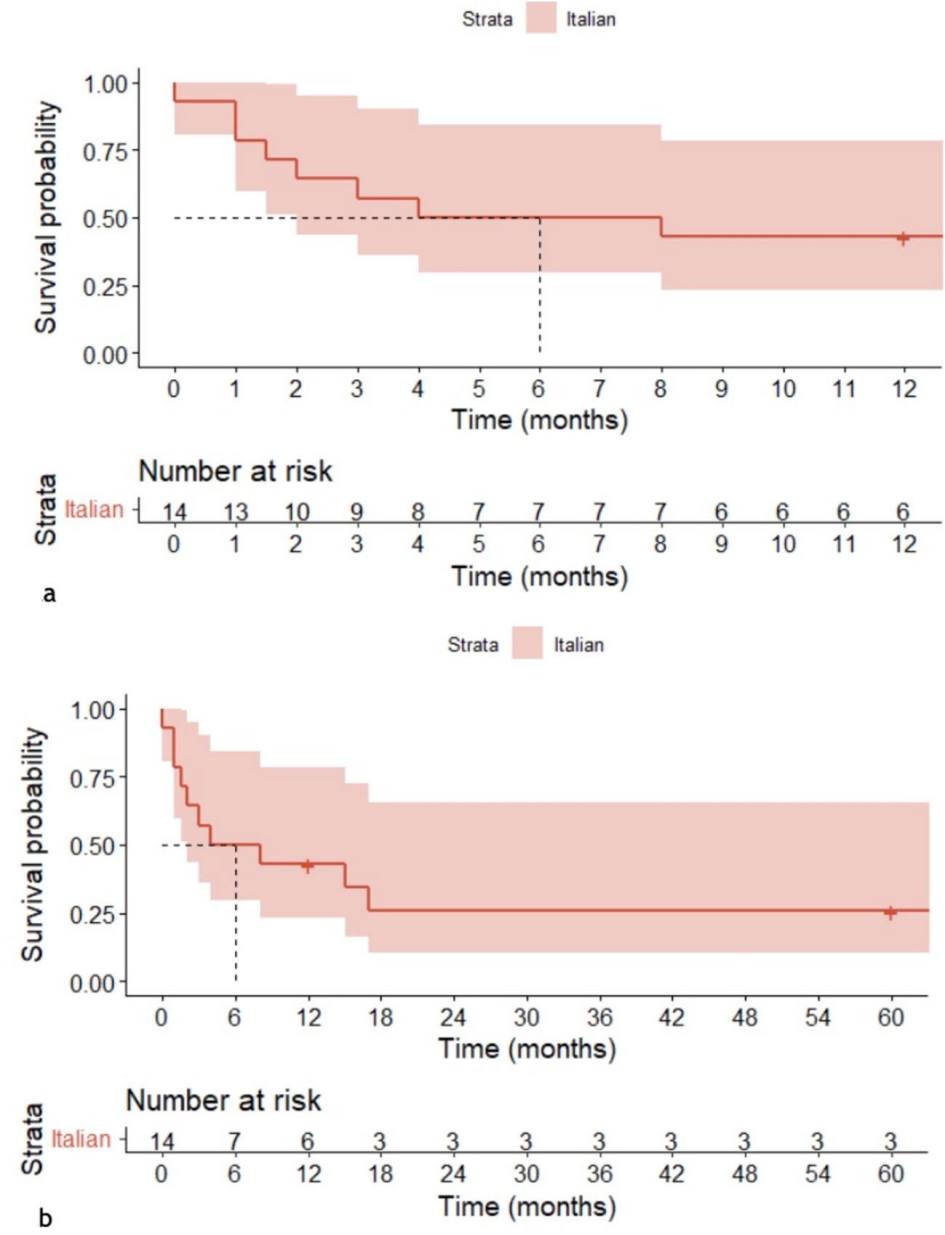
One-year survival (**a**) and 5-year (**b**) survival plots for the Italian cohort

**Fig. 3:**
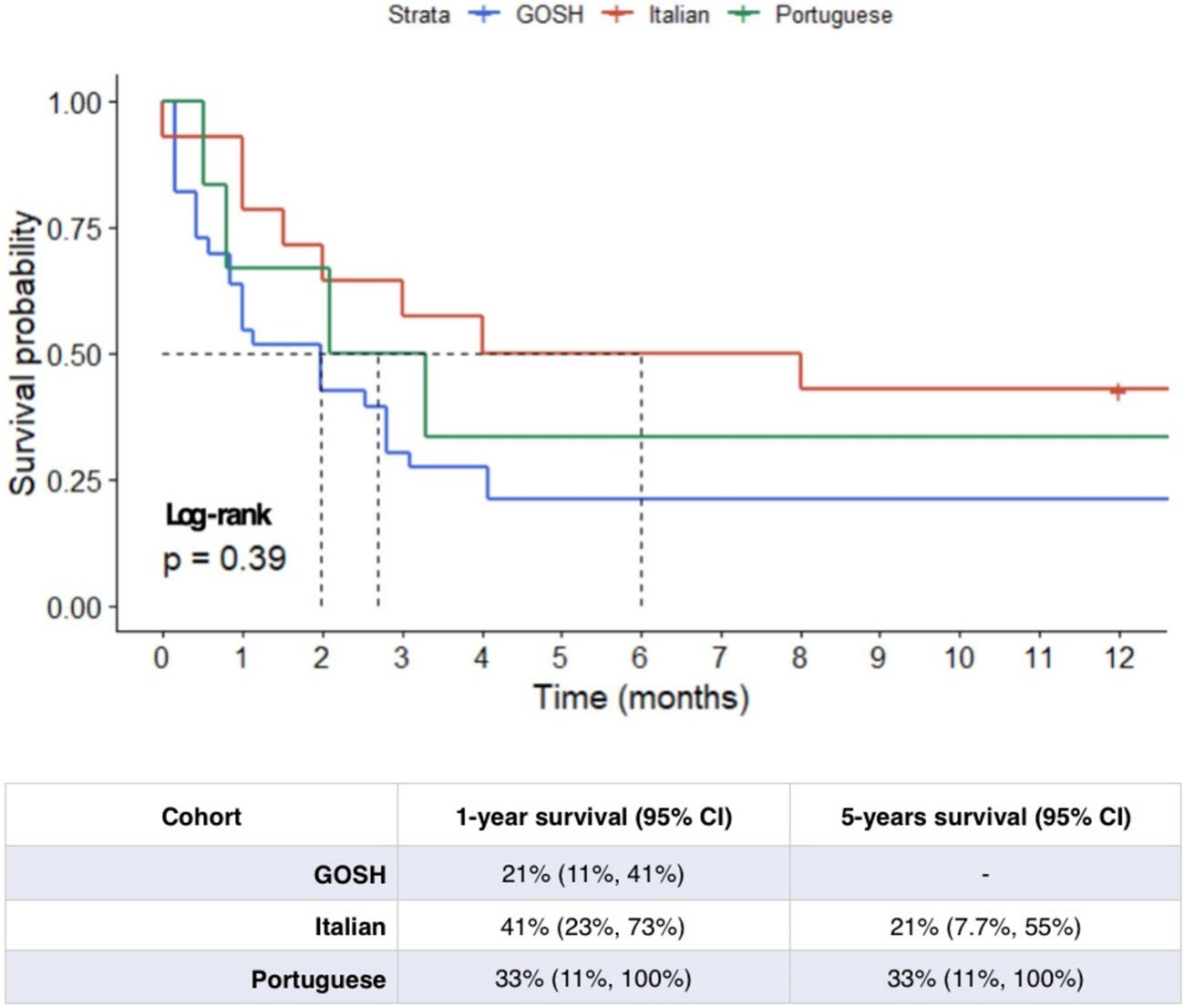
Comparison of survival plot between Italian-study cohort and three other cohorts reported by recent literature (the Portuguese, Great Ormond Street Hospital (GOSH), and Canadian cohorts) [6, 10, 20], reconstructed after data extraction from the reference publications. In red the Italian Cohort, in blue and green the GOSH and Portuguese cohorts. The dotted lined indicate the median survival for each of the cohorts. The result of the log-rank test indicates the absence of important differences between the investigated cohorts

Figure 2 reports 1- and 5-year survival plots for this Italian cohort. The 5-year survival curve for the Italian cohort shows an overall 5-year survival probability of 25% for the subjects included in the study. This has been compared to some cohorts reported by recent literature (the Portuguese, UK and Canadian cohorts) [6, 10, 20], reconstructed after data extraction from these reference publications. The 1-year survival rate is 43% for the Italian cohort, compared to 21% (95% CI: 11–41%) for the UK cohort, 33% (95% CI: 11–100%) for the Portuguese cohort, and 12% for the Canadian cohort (95%CI: 8.5%, 17%) (Fig. 3). Dashed lines indicate median survival for each of the cohorts. The result of the Gehan-Breslow test is not significant when we consider the Italian, Portuguese, and UK cohorts (*p* value: 0.19); the corresponding *p* value decreases (*p* value: < 0.001) only when including the Canadian cohort, as the test used gives more weight to deaths at early time points (Fig. 2), which characterize the latter cohort [10]. In the case of the Italian cohort, approximately 12% of the patients received at least one surgical operation. In the UK cohort, at least 8% of the children underwent surgery [20]. Finally, approximately 33% of subjects were operated on in the Portuguese cohort [6] and 13.8% of subjects in the Canadian cohort [10]. Despite the different percentages, the survival trend in the European cohorts does not seem to differ significantly in its shape after the initial phases, characterized by relevant perinatal mortality. It could indicate how performing surgical interventions may not alter the clinical course of the disease. More data needs to be collected to confirm or refute this trend.

## Discussion

Over the previous 15 years, 17 children with T13 and T18 have been followed by our regional PPC center. The great majority of defects reported are consistent with the standard phenotypes of T13 and T18. All patients were born with intrauterine growth restriction, with a median GA of 36 weeks and BW 3°, as previously observed [12–14]. Our cohort’s patients all had a heart anomaly; however, just two (12%) received cardiac surgery. This statistic is consistent with previous research in which 7–26% of the children underwent heart surgery [3, 20–22].

Recent studies show an increase in major surgical treatments for patients with T13 and T18, as corrective treatment for congenital anomalies such as tracheostomy, gastrostomy, and cardiac surgery [3, 20–22]. Some authors report on how these measures can help these children live longer lives [23, 24]; however, clear assessments of long-term outcomes and comorbidities other than death in children with T13 or T18 are sparse and frequently given only as case reports or series [25–27]. Other authors have discussed how cardiac surgery can help with home care transition [27, 28], reduce in-hospital mortality, and make home care more accessible [24]. Furthermore, James D. et al. developed recommendations for surgical intervention in CHD patients, evaluating each individual case based on the presence and severity of comorbidities [28].

Mizrah’s research also suggests that a considerable number of hospitalizations in the cardiac surgery group occurred prior to their congenital heart surgery [29], although data for the post-surgery period are scarce. Moreover, newborns with T13 and T18 who require critical care units and mechanical ventilation on their first day of birth may have poor post-operative outcomes following CHD surgery [3, 4]. According to data reported in a prior study [27], the two infants in our cohort who underwent major heart surgery and tracheostomy experienced a significant delay in discharge compared to the other patients, who achieved initial home discharge within the first 6 weeks of life. Children in our study group who had major surgery had more than two hospitalizations per year, with a single case reaching a record high of 12, due to recurrent infections. According to the literature [29, 30], this group of patients had a longer and higher hospitalization rate when compared to patients who did not have any surgery, regardless of the degree of CHD, as well as a higher rate of gastrostomy tube placement and tracheostomy [30, 31].

Despite only two children receiving heart surgery, and with the exception of two newborns with T18 who died within the first 24 h of birth, all patients in our cohort achieved home care, regardless of surgical status or type of CHD. If gastrostomy was not performed to manage feeding intolerance, all patients were discharged with a nasogastric tube, and in four cases, gastrostomy was performed after the first 6 months of life with the parents’ and PPC team’s agreement, because this intervention was valued as useful and fundamental for the child in order to increase QoL. All patients discharged to home received an aspiration device and a pulse oximeter, and all parents were instructed to use these devices prior to being discharged by the PPC team. This data reveals that domiciliation is not connected to the degree of complexity of the child’s care, but rather relies on the type of service provided by each PPC team. The primary variables that enabled release to home were the use of home care and a full re-organization of local healthcare services as the possibility to have punctual visit by home care, as well as investments in parental training, educational assistance, and the presence of healthcare professionals, including nurses and home hospice support, to sustain families.

The research goal of identifying valuable outcomes in this patient cohort should include not only the survival rate, the decision whether to perform major surgical interventions, but also the true quantification of QoL for children and families, which is an important missing point [32]. Patient-oriented outcomes are not often quantified in the short and midterm. We tried to estimate family member’s QoL for alive patients with FROM16. Median of the emotional score was 1/12 (0–4) items and 12/20 [12, 13] about personal and social life with a total of 13/34 [14–17]. These values are in line with other FROM16 scores reported by family members of patients with chronic diseases (mean = 12.3, SD = 7.5, median = 11.50, *n* = 120, *p* < 0.001) [9], but they are still lower than family members of neurology patients (19.8), oncology patients (17.6), hematology patients (16.6), and chronic pain patients (16.6) [18]. This tool could be more widely utilized, as it is the first generic family QoL measure which may potentially be used in clinical situations to monitor the evolution of QoL impact and could encourage clinicians to focus on the family’s QoL for children with complex healthcare. Another unanswered concern is whether accurate risk categorization of these patients is achievable in terms of assessment of potential complications and progression of each organ insufficiency early in infancy, which is currently lacking. We have many reports on the overall survival of these children and the natural history of the disease, but many reports published with a large cohort, such as Wu et al. [32], do not specify what type of intervention is offered to their patients and only focus on genetic characteristics. The literature suggests that T18 mosaicism has a higher chance of survival [32], but the impact of surgical and nonsurgical therapies in the context of rehabilitation services and family assistance with home care is not described. The presence of CHD and the type of CHD appears to be one factor determining patient prognosis, as Rasmussen et al. [33] showed that children without heart problems had a greater 1-year survival rate than those with heart defects.

From an ethical perspective, as management and treatment options for T13 and T18 evolve, we must consider whether any intervention we offer for a single-organ insufficiency, when considered in the context of multiple comorbidities associated with the genetic diagnosis, is reasonable for the child and their family. The prospect of performing surgical interventions and providing medical therapies for these children must be explored with a tailored view of the patient’s care, while also considering the patient’s QoL as well as his family’s overall values, goals, and QoL [9, 34–36]. According to different organizations of healthcare systems around the world, some face varying access to care. The PPC team cannot be represented in certain countries [24] or in others (where the healthcare system is linked to insurance companies); socioeconomic variations can influence the possibility for significant disparities in patient treatment [29].

This study has several limitations, as it is restricted to a single-center experience. Our cohort is very selected, both statistically and in terms of features, as it represents a group of patients who have been attended by the PPC team immediately in the perinatal period, even if not all of them were at the prenatal level. The Canadian cohort was gathered between 1991 and 2012, making it only partially similar to the three European courts studied, which are more recent and may indicate a different approach to patient management. Many aspects related to the management and transition from hospital to home care are not detailed, and this makes it difficult to compare the cohorts at this nuanced level. The most interesting aspect of survival curves is that despite the fact that differing proportions of patients underwent surgical treatments, this does not appear to alter medium-long-term survival beyond the fifth year. More multicentric data should be collected in order to capture families’ views and needs while also attempting to standardize home care support and give a uniform strategy at least on a national level [35]. As a result, our reported survival curve may not give a complete picture of survival outcomes, and readers should interpret our findings with caution. Additionally, we did not have any patient data on T18 mosaicism.

Future studies should be oriented toward understanding barriers to discharge from the hospital as an example of patient-oriented outcome in order to understand which intrinsic factors, such as maternal and perinatal factors, genetic factors, and comorbidities, affect discharge home. Additionally, social and family conditions are an important part of the risk assessment for the first weeks of life and for the local support team organization. Early deployment and referral to specialist PPC teams are critical strategies for tackling this issue. From prenatal and delivery preparations through postnatal care, these teams can provide new views and insights to support the most suitable treatment throughout the lifespan.

## Conclusion

There are still numerous unanswered concerns for healthcare practitioners involved in T13 and T18 treatment. In order to focus suitable interventions in future outcome studies, the possibility of early severe evolution of medical complexity must be addressed as a primary driver. Clinical and medical complexity, as well as patient outcomes, are intrinsically linked to resource availability and home care programs.

## Data Availability

No datasets were generated or analysed during the current study.

## Abbreviations

ACCAPED: Scheda di ACcertamento dei bisogni Clinico Assistenziali complessi in PEDiatria
ACC: Agenesis of Corpus Callosum
AP window: Aorto-pulmonary window
ARF: Acute respiratory failure
AVC: Atrioventricular canal defect
BAV: Bicuspid aortic valve
BW: Birth weight
CA: Cardiac arrest
CHD: Congenital heart disease
CPCs: Choroid plexus cysts
CVH: Cerebellar vermis hypoplasia
DORV: Double outlet right ventricle
DNR: Do Not Resuscitate
FROM-16: Family Reported Outcome Measure-16
GA: Gestational age
HAA: Hypoplastic aortic arch
HF: Heart failure
LB: Live birth
mPA enlargement: Main pulmonary artery enlargement
PAD: Pendant arterial defect
PPC: Pediatric palliative care
PFO: Patent foramen ovale
QoL: Quality of life
SB: Still birth
T13: Trisomy 13
T18: Trisomy 18
VSD: Ventricular septal defect
