# Correction to: Early assessment of clinical complexity and home care in patients affected by trisomy 13 and 18

**DOI:** 10.1007/s00431-025-06419-8

**Published:** 2025-09-19

**Authors:** Anna Zanin, Matteo Patti, Isabella Rosato, Antuan Divisic, Francesca Rusalen, Irene Maghini, Caterina Agosto, Franca Benini

**Affiliations:** 1 Palliative Care and Pain Service, Department of Women’s and Children’s Health, University of Padua, Via Giustiniani 3, 35128 Padua, Italy; 2 Department of Women’s and Children’s Health, University of Padua, Padua, Italy; 3 Unit of Biostatistics, Epidemiology and Public Health, Department of Cardio-Thoraco-Vascular Sciences and Public Health, University of Padua, Padua, Italy

**Correction to: European Journal of Pediatrics (2025) 184:194**

10.1007/s00431-025-06020-z

In the originally published article entitled "Early assessment of clinical complexity and home care in patients affected by trisomy 13 and 18," the Tables 4 and 5 contains errors. In the original publication, incorrect data values were presented in these tables, leading to a misrepresentation of the DNR (Do Not Resuscitate) choices within the study cohort. The original data in Tables 4 and 5 are now recognized as inaccurate.

**Incorrect Tables:**

The following tables contained errors:

Table 4 Descriptive analysis of the Italian cohort’s complexity of care

**Table:** 

		Italian cohort
Median number of medical devices (range), *n* = 16		3 (0–5)
Median number of drugs taken daily (range), *n* = 8		4 (1–10)
Polypharmacy (%), *n* = 8	Yes	3 (38%)
	No	5 (63%)
Median ACCAPED (range), *n* = 17		86 (38–129)
Median number of hospitalizations in the last year (range), *n* = 15		1 (0–12)
Median number of procedures performed (range), *n* = 17		0 (0–3)
DNR (%), *n* = 16	Yes	6 (38%)
	No	10 (63%)
Median FROM 16 Emotional (range), *n* = 3		1 (0–4)
Median FROM 16 Personal (range), *n* = 3		12 (11–12)

Polypharmacy described as ≥ 5 drugs/daily; medical device list includes aspirator, nasogastric tube, tracheostomy, enteral nutrition pump, pulse oxymeter

*ACCAPED* scheda di ACcertamento dei bisogni Clinico Assistenziali complessi in PEDiatria, *DNR* do not resuscitate order, *FROM-16* Family Reported Outcome Measure-16

Table 5 Complexity of care of alive and deceased patients

**Table:** 

		Total *N* *=* *17*	Alive *N* *=* *4*	Deceased *N* *=* *13*	*p* value*
Median number of medical devices (range), *n* = 16		3 (0–7)	4.5 (2–7)	3 (0–5)	0.248
Number of drugs taken daily (*n*, %), *n* = 17		9 (53%)	1 (25%)	8 (62%)	0.039
0		3 (18%)	0 (0%)	3 (23%)	
1–3		3 (18%)	1 (25%)	2 (15%)	
4–6		2 (12%)	2 (50%)	0 (0%)	
7–10					
Polypharmacy (n, %), *n* = 17	Yes	3 (18%)	2 (50%)	1 (8%)	0.052
	No	14 (82%)	2 (50%)	12 (92%)	
Median ACCAPED (range), *n*=17		86 (38–129)	86 (38–129)	87 (38–104)	0.955
Patients who were hospitalized 1 + times in the last year (*n*, %), *n* = 15	Yes	10 (66.7%)	2 (50%)	8 (73%)	0.409
	No	5 (33.3%)	2 (50%)	3 (27%)	
DNR (%), *n* = 16	Yes	6 (38%)	2 (12.5%)	4 (25.0%)	0.551
	No	10 (63%)	2 (12.5%)	8 (50.0%)	

Polypharmacy described as ≥ 5 drugs/daily, medical devices

*ACCAPED* scheda di ACcertamento dei bisogni Clinico Assistenziali complessi in PEDiatria, *DNR* do not resuscitate order, *FROM-16* Family Reported Outcome Measure-16

^a^Mann-Whitney test or Fisher’s exact test according to variables investigated

**Corrected Tables:**

The corrected Tables 4 and 5 are presented below.

**Table 4. Descriptive analysis of the Italian cohort’s complexity of care**

**Table:** 

		**Italian cohort**
**Median number of medical devices (range), n = 16**		3 (0–5)
**Median number of drugs taken daily (range), n = 8**		4 (1–10)
**Polypharmacy (%), n = 8**	*Yes* *No*	3 (38%) 5 (63%)
**Median ACCAPED (range), n = 17**		86 (38–129)
**Median number of hospitalizations in the last year (range), n = 15**		1 (0–12)
**Median number of procedures performed (range), n = 17**		0 (0–3)
**DNR (%), n = 17**	*Yes* *No*	13 (76%) 4 (23%)
**Median FROM 16 Emotional (range), n = 3**		1 (0–4)
**Median FROM 16 Personal (range), n = 3**		12 (11–12)

**Table 5. Complexity of care of alive and deceased patients**

**Table:** 

		**Total** **N = 17**	**Alive** **N = 4**	**Deceased** **N = 13**	p-value*
**Median number of medical devices (range), n = 16**		3 (0–7)	4.5 (2–7)	3 (0–5)	0.248
**Number of drugs taken daily (n, %), n = 17**					
*0* *1–3* *4–6* *7–10*		9 (53%) 3 (18%) 3 (18%) 2 (12%)	1 (25%) 0 (0%) 1 (25%) 2 (50%)	8 (62%) 3 (23%) 2 (15%) 0 (0%)	0.039
**Polypharmacy (n, %), n = 17**	*Yes* *No*	3 (18%) 14 (82%)	2 (50%) 2 (50%)	1 (8%) 12 (92%)	0.052
**Median ACCAPED (range), n = 17**		86 (38–129)	86 (38–129)	87 (38–104)	0.955
**Patients who were hospitalized 1 + times in the last year (n, %), n = 15**	*Yes* *No*	10 (66.7%) 5 (33.3%)	2 (50%) 2 (50%)	8 (73%) 3 (27%)	0.409
**DNR (%), n = 17**	*Yes* *No*	13 (76%) 4 (23%)	2 (12.5%) 2 (12.5%)	11 (85%) 2 (15%)	0.22

*Mann-Whitney test or Fisher’s exact test according to variables investigated

**Impact of the Error:**

The incorrect data in Tables 4 and 5 resulted in a misinterpretation of the DNR choice patterns within the study cohort. The correct data, presented in the corrected tables, provide a more accurate representation of the patient choices. The results and discussion section of the original article correctly reflect the data from the corrected tables.

**Conclusion:**

The authors sincerely apologize for the errors in Tables 4 and 5. The corrected tables are provided for clarity and accuracy. The original conclusions remain valid when considering the corrected data.

The original article has been corrected.

